# From photos to sketches - how humans and deep neural networks process objects across different levels of visual abstraction

**DOI:** 10.1167/jov.22.2.4

**Published:** 2022-02-07

**Authors:** Johannes J. D. Singer, Katja Seeliger, Tim C. Kietzmann, Martin N. Hebart

**Affiliations:** 1 Vision and Computational Cognition Group, Max Planck Institute for Human Cognitive and Brain Sciences, Leipzig, Germany; 2 Department of Psychology, Ludwig Maximilian University, Munich, Germany; 3 Donders Institute for Brain, Cognition and Behavior, Nijmegen, The Netherlands

**Keywords:** deep convolutional neural networks, generalization, drawings, representational similarity analysis

## Abstract

Line drawings convey meaning with just a few strokes. Despite strong simplifications, humans can recognize objects depicted in such abstracted images without effort. To what degree do deep convolutional neural networks (CNNs) mirror this human ability to generalize to abstracted object images? While CNNs trained on natural images have been shown to exhibit poor classification performance on drawings, other work has demonstrated highly similar latent representations in the networks for abstracted and natural images. Here, we address these seemingly conflicting findings by analyzing the activation patterns of a CNN trained on natural images across a set of photographs, drawings, and sketches of the same objects and comparing them to human behavior. We find a highly similar representational structure across levels of visual abstraction in early and intermediate layers of the network. This similarity, however, does not translate to later stages in the network, resulting in low classification performance for drawings and sketches. We identified that texture bias in CNNs contributes to the dissimilar representational structure in late layers and the poor performance on drawings. Finally, by fine-tuning late network layers with object drawings, we show that performance can be largely restored, demonstrating the general utility of features learned on natural images in early and intermediate layers for the recognition of drawings. In conclusion, generalization to abstracted images, such as drawings, seems to be an emergent property of CNNs trained on natural images, which is, however, suppressed by domain-related biases that arise during later processing stages in the network.

## Introduction

Humans have the remarkable ability to robustly recognize objects across a wide range of visual abstractions. One striking example for this feat is that we can identify objects in simple and abstract line drawings with similar speed and accuracy as natural object images (Biederman & Ju, 1988; Eitz, Hays, & Alexa, 2012). Neuroimaging studies have shown that this behavior is supported by a similar neural representation for natural images and line drawings across a number of visually responsive brain regions (Ishai, Ungerleider, Martin, Schouten, & Haxby, 1999; Walther, Chai, Caddigan, Beck, & Fei-Fei, 2011). This suggests that both the recognition of natural images and line drawings of objects rely on a general-purpose neural architecture that supports general object recognition. Recent developments in computer vision and computational neuroscience have opened up new avenues for studying the computational mechanisms of object recognition in silico (Kietzmann, McClure, & Kriegeskorte, 2019), with deep convolutional neural networks (CNNs) as the most successful and most popular model class in approximating the activity elicited during object recognition in the inferior temporal cortex of non-human primates and humans (Cichy, Khosla, Pantazis, Torralba, & Oliva, 2016; Khaligh-Razavi & Kriegeskorte, 2014; Storrs, Kietzmann, Walther, Mehrer, & Kriegeskorte, 2020; Yamins, Hong, Cadieu, Solomon, Seibert, & DiCarlo, 2014). While CNNs have yielded important insights into the mechanistic underpinnings of visual object recognition and show impressive performance in object classification benchmarks (Krizhevsky, Sutzkever, & Hinton, 2012; Simonyan & Zisserman, 2015), there is a limited understanding of how well the recognition abilities of CNNs generalize to abstracted visual stimuli such as line drawings. Identifying the extent to which CNNs generalize to drawings could therefore not only deepen our understanding of the inner workings of CNNs themselves, but also create an important benchmark case for comparing CNNs and biological vision.

Recent studies investigating human-like generalization to drawings in CNNs have yielded conflicting results. On the one hand, there is evidence that CNNs trained on natural images cannot recognize abstract drawings and achieve poor classification performance with such images (Ballester & Araujo, 2016; Evans, Malhotra, & Bowers, 2021; Wang, Ge, Xing, & Lipton, 2019). This might be attributed to texture bias in CNNs, referring to the recent demonstration that CNNs tend to rely more strongly on texture rather than shape information for object recognition, which contrasts with humans who rely more strongly on shape (Geirhos, Janssen, Schütt, Rauber, Bethge, & Wichmann, 2019; Hermann, Chen, & Kornblith, 2020). Since the texture of drawings is altered strongly in respect to photographs (in the following “photos”, for brevity), CNNs trained on the latter may no longer recognize them without this diagnostic information. On the other hand, notable performance on colored and highly detailed drawings has been shown (Kubilius, Bracci, & Op de Beeck, 2016), and others have argued for a common representational format for drawings and photos in CNNs trained on natural images (Fan, Yamins, & Turk-Browne, 2018). This can be explained with findings showing that CNNs, too, accurately capture abstract shape information in their representational spaces (Kalfas, Vinken, & Vogels, 2018; Kubilius et al., 2016). Since the overall shape of an object is largely preserved in drawings, based on these findings it might be expected that the representations and performance for drawings are similar to those found for natural photos. Together, these seemingly disparate findings and their underlying explanations point to a gap in our understanding of the emergence of generalization to drawings in CNNs.

Here, we address this gap by assessing the extent to which CNNs trained on natural images process object images similarly across different levels of visual abstraction. To this end, we composed a set of images of the same objects across three levels of visual abstraction: natural photos, line drawings, and sketches. In addition to analyzing the classification performance of the networks on these images, we compare the internal representations in the networks to each other and to human similarity judgments by using representational similarity analysis (Kriegeskorte, Mur, & Bandettini, 2008).

## Methods

### Code

We made all code used for the present analyses publicly available (https://github.com/Singerjohannes/object_drawing_DNN). The analysis of the network's performance as well as feature extraction were implemented using Python version 3.6.9 and the PyTorch version 1.6.0 API (Paszke, Gross, Massa, Lerer, Bradbury, & Chanan, 2019). Further analyses based upon the network activations and the human behavioral data were implemented using MATLAB R2017a (www.mathworks.com).

### Stimuli

**Figure 1.**
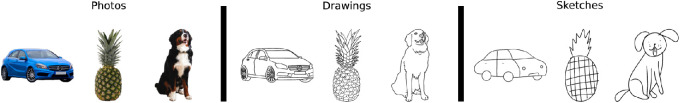
**Examples from the stimulus set used in all experiments.** We created a stimulus set of 126 stimuli originating from 42 object categories (21 manmade and 21 natural) depicted across three levels of visual abstraction. The three types contained the same objects as natural photos cropped from their background (“photos”), line drawings (“drawings”), and sketch-like drawings (“sketches”).

Our stimulus set comprised 42 object categories (21 manmade and 21 natural), each depicted across three different types of depiction: “photos,” “drawings,” and “sketches” (126 stimuli total; Figure 1). Drawings and sketches were drawn by a professional artist for the purpose of this study. For the first type of depiction (“photos”), object-images were natural photos of objects, cropped from their background. For the second type of depiction (“drawings”), we gathered line drawings of the same objects. For these line drawings, color and texture information was strongly altered, while contour-level information was highly similar to the photos. For the third type of depiction (“sketches”), the images lacked even more detail as compared to the photos, with distortions in both the contours and the size of some features, rendering them physically even more dissimilar to the photos.

### Models

For all experiments, we used the VGG-16 architecture (Simonyan & Zisserman, 2015), which won the ILSVRC-challenge in the year 2014 with a top-5 accuracy of 92.7% on the test set. The network was chosen for its relative simplicity compared to deeper and more complex network architectures and for its documented similarities to visual processing in the human brain (Güçlü & van Gerven, 2015; Jozwik, Kriegeskorte, Storrs, & Mur, 2017; Kalfas et al., 2018; Nonaka, Majima, Aoki, & Kamitani, 2020; Schrimpf, Kubilius, Hong, Majaj, Rajalingham, Issa, et al., 2020; Storrs et al., 2020). VGG-16 is a 16-layer network which contains five convolutional blocks, each followed by a max-pooling layer. After the convolutional stage of the network, there are three fully connected layers, with the last layer being the softmax layer, which outputs the probabilities for the 1000-way classification task. For experiment 1, we used the standard implementation of VGG-16 pretrained on the ILSVRC2012 dataset (Russakovsky, Deng, Su, Krause, Satheesh, Ma, et al., 2015) without batch normalization. For simplicity, in the following, we will refer to networks trained with the ILSVRC2012 dataset as “ImageNet-trained.” In addition, we evaluated a variant of VGG-16 in experiment 1 that was not pretrained and was initialized with random weights. For experiment 2, we obtained the version of VGG-16, which was trained on stylized ImageNet – a modified version of the ImageNet dataset (https://github.com/rgeirhos/Stylized-ImageNet). Stylized ImageNet was created using image style transfer (Gatys, Ecker, & Bethge, 2016), with the goal of making shape a more diagnostic feature than texture in the training data (Geirhos, Rubisch, Michaelis, Bethge, Wichmann, & Brendel, 2019). For experiment 3, we used the same standard VGG-16 implementation as in experiment 1 but carried out fine-tuning on the weights of the layers (for details, see below).

### Image preprocessing

Before passing the images through the network, they were scaled and placed on a square grey background. Further, the images were resized to the input size of 224 × 224 and normalized (zero mean, unit variance).

### Performance evaluation

In order to quantify the performance of the networks in our experiments, we computed the top-1 accuracy based on the outputs of the softmax layer of the models. We mapped the categories in our stimulus set to the ImageNet categories by using the WordNet 3.0 hierarchy (Miller, 1995). A response was counted as correct if the highest scoring label matched the given category label or any of its hyponyms in the WordNet 3.0 hierarchy (Miller, 1995). For example, for the category “dog,” each response was counted as correct that referred to any of the labels in the ImageNet classes that shared the hypernym “domestic dog” in the WordNet 3.0 hierarchy (Miller, 1995). While this approach may slightly overestimate the networks’ overall classification performance, it was held constant for all relevant comparisons and thus should not affect the overall interpretation of results.

### Feature extraction

We extracted the activation patterns for all stimuli only from the five pooling layers and the first two fully connected layers but excluded the softmax layer from our analyses. For comparing activations in pooling layers, we flattened the extracted feature map activations.

### Fine-tuning

The VGG-16 model was fine-tuned using the ImageNet-Sketch data set (Wang et al., 2019). ImageNet-Sketch consists of approximately 50 training examples of drawings for each of the 1000 ImageNet classes, resulting in approximately 51,000 training images of drawings. It is important to note that ImageNet-Sketch is orders of magnitude smaller than the ILSVRC2012 data set (1.4 million examples from ImageNet; Deng, Dong, Socher, Li, Kai, & Li, 2009) that has been used for training the original VGG-16 network. For the fine-tuning procedure, layers conv1-1 to conv4-3 were frozen, and only the last convolutional layers conv5-1 to conv5-3 and the fully connected layers were adjusted. The model weights of those layers were then trained using the stochastic gradient descent (SGD) optimizer. A hyperparameter search for the learning rate and momentum was performed. With the optimized learning rate of 0.001 and momentum of 0.7, a top-1 accuracy of 66.35% was reached on a validation set of 2000 images.

### Behavioral tasks

All human behavioral data were acquired in online experiments using the Amazon Mechanical Turk Platform (Mturk). All participants were located in the United States. In the labeling task, 480 individuals (280 women, 196 men, and 4 other) took part, with a mean age of 37.96 years (*SD* = 12.45). In the triplet task 742 (404 women, 334 men, and 4 other) participants took part, with a mean age of 38.29 years (*SD* = 12.54). The experimental protocols were approved by the local ethics committee (012/20-ek) in accordance with the Declaration of Helsinki, and all participants provided informed consent.

#### Labeling task

To measure the accuracy of humans in recognizing our stimuli, we conducted an image labeling experiment. In this task, workers on Mturk were instructed to give a label for a given image consisting of one word, as if they were trying to find the corresponding entry for the given image in a dictionary. Labels were regarded correct if the label matched the category label of the object exactly or if the label was a synonym for the given category label. In order to avoid carryover effects between types of depiction, all trials in a given task were limited to one depiction type, and a given participant could only participate in one task.

#### Triplet task

To measure the perceived similarity of objects in our image sets, we conducted a triplet odd-one-out similarity task (Hebart, Zheng, Pereira, & Baker, 2020). For each given trial, participants were presented with images from three different object categories side by side. Triplets always consisted of the same type of image depiction. Participants were instructed to indicate which of the three object images in the trial they thought is the odd-one-out by clicking on the respective image using their computer mouse or touchpad. In order to minimize experimenter bias, there were no instructions as to what strategy they should use to find the odd-one-out, meaning that they could use any information as the basis of their judgment which seemed relevant to them. Additionally, if they did not recognize the object, participants were instructed to base their judgment on their best guess of what the object could be. As in the labeling task, images in one task were limited to one type of depiction, and participants could only work on one task. We subsequently computed the object similarity between a given object pair for one type of depiction in our stimulus set as the probability of choosing objects x and y to belong together, across all participants’ ratings. We calculated this probability as the ratio of trials in which objects x and y were presented together with different objects z in the triplet when object z was chosen as the odd-one-out. Therefore, the probability of choosing objects x and y together reflects the perceived similarity between objects x and y, irrespective of the context imposed by the third object z in the triplet.

### Quantifying representational similarity across types of depiction

**Figure 2.**
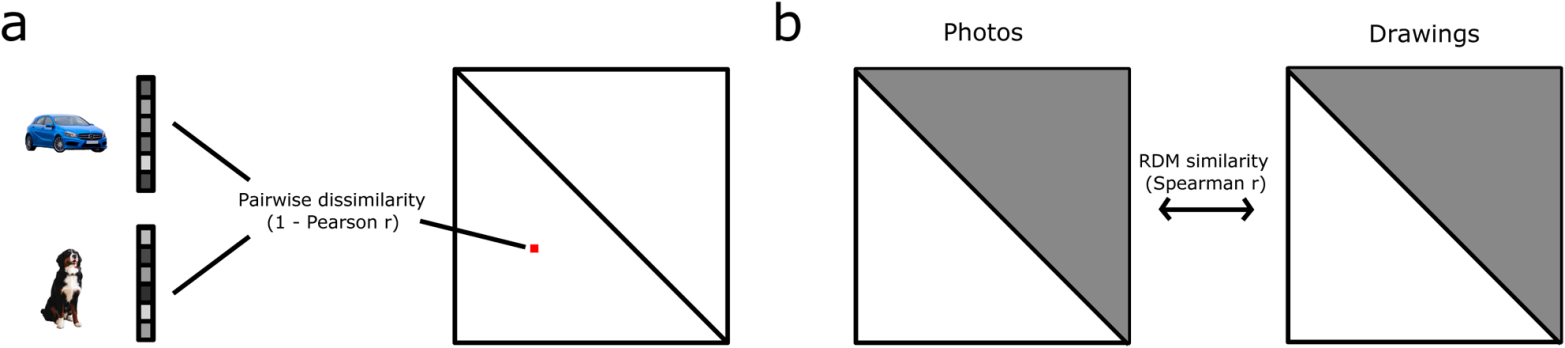
**Schematic of the RSA approach used in all experiments.** (**A**) For analyzing the internal representations of the networks, we extracted the activation patterns to the stimuli in our set from a selection of layers. Subsequently, we computed RDMs based on the correlation distances (1 - Pearson *r*) between all pairs of stimuli in one type of depiction and for each given layer separately. This yielded three RDMs (one for each type of depiction) in every selected layer. (**B**) In order to obtain a measure of similarity across types of depiction regarding the internal representation in the networks, we computed the Spearman rank correlation between the lower triangular values of RDMs of the different types of depiction in each layer separately. For the RDMs comparing representations of photos and drawings, this yields a “photo-to-drawing” similarity.

We used representational similarity analysis (RSA; Kriegeskorte et al., 2008) to quantitatively describe the representational object space in the CNNs in our experiments as well as in human behavioral measurements. RSA characterizes representations by estimating the geometry of stimulus responses in high-dimensional population space. For this, the similarity of all pairwise comparisons of conditions in the experimental design is computed by comparing responses of a given system (e.g. activation patterns extracted from a CNN) for every pair of conditions and arranging them in a matrix format. The matrix of dissimilarities is commonly referred to as a representational dissimilarity matrix (RDM). The power of RSA lies in the fact that RDMs can be computed and subsequently compared across different systems (e.g. computational models and humans) but also across different conditions and processing stages in a neural network (Mehrer, Spoerer, Kriegeskorte, & Kietzmann, 2020). As our stimulus set contained the same object categories for each type of depiction, we could directly compare the RDMs across different types of depiction by correlating the RDMs with each other. We computed RDMs based on the activation patterns that we extracted from the networks separately for each type of depiction and for each selected layer. We then calculated the Spearman rank correlation between the RDM's lower triangular parts to acquire a measure of similarity across types of depiction in terms of the network's internal representation (Figure 2).

#### Super RDMs and multidimensional scaling

For comparisons of the representational geometries of all three types of depiction combined, we constructed super RDMs based on the activation patterns of the responses of the network to all stimuli in the set following the RSA procedure described above. For visualization purposes, we projected the RDMs into a two-dimensional space using multidimensional scaling (metric stress).

### Classification analysis

#### Intra-domain classification

To quantify the information contained in the activation patterns of the networks in a given layer, we performed a classification analysis on the extracted activation patterns. To this end, we trained a linear Support Vector Machine classifier (Chang & Lin, 2011) on the activation patterns to distinguish between patterns of manmade and natural objects (c = 1). We followed a leave-*N*-out cross-validation framework (*N* = 6), meaning that we trained the classifier on all but a left-out sample of six activation patterns and evaluated the accuracy of the classifier's prediction on the left-out sample. We repeated this procedure 1000 times for a given layer while randomly drawing training and test examples from all activation patterns according to the framework described above. Finally, we averaged the accuracies over all 1000 iterations, yielding mean classification accuracies and standard errors for each layer and each type of depiction in the network.

#### Cross-domain classification

In an additional analysis step, we trained the classifier following the same procedure as described above but used the activation patterns of one type of depiction (e.g. photos) and tested the classifier on the activation patterns of another type of depiction (e.g. drawings).

### Statistical analyses

To statistically evaluate differences in classification performance between networks or between types of depiction in one network, we used the McNemar test of homogeneity (McNemar, 1947). Furthermore, to test for differences between levels of human performance, we used a two-sided independent *t*-test. For testing the equivalence of the means in human behavioral accuracies, we followed a two one-sided tests procedure, as described in (Lakens, 2017). For all other comparisons, we used a one-sided randomization testing procedure. This procedure entailed computing a null distribution by repeated randomization and subsequently obtaining the *p* value by finding the percentile of values in the null distribution that reaches or exceeds the empirical value. For comparing human performance with CNN performance, we obtained the null distribution using a sign permutation procedure. To do this, we permuted the signs of human accuracies for single objects for a given type of depiction, subtracted the CNN accuracy from the permuted human accuracies, averaged the accuracies, and repeated this procedure 1000 times. To test for the statistical significance of a given RDM-correlation, we randomly shuffled object labels for one RDM, computed the correlation with the reference RDM, and repeated this procedure 1000 times to obtain a null distribution (Mantel test; Mantel, 1967). To test for changes in representational similarity across layers (e.g. for “photos” versus “drawings”), we randomly shuffled object labels, using the same shuffling across layers but a different shuffling for different types of depiction. Then we created permuted RDMs and subsequently computed the sum of squared differences between the overall mean in RDM correlations across layers and the individual RDM correlations. We then repeated this procedure 1000 times to obtain a null distribution of sum of squared differences. Furthermore, to test for pairwise differences between two given RDM correlations, we created permuted RDMs for one of the RDMs from both correlations by shuffling the object labels in one of the correlated RDMs. We then computed the correlation with these permuted RDMs and subtracted the two correlation values from each other. By repeating this procedure 1000 times we obtained a null distribution of the difference between two RDM correlations. Finally, in order to test if a given classification accuracy significantly exceeded chance level, we obtained the null distribution by repeating the classification analysis 1000 times with randomly shuffled labels.

#### Correction for multiple testing

For all statistical tests reported here, we corrected the *p* values for multiple comparisons by using the Benjamini-Hochberg false discovery rate correction (Benjamini & Hochberg, 1995).

## Results

### Experiment 1: Generalization to drawings in an ImageNet-trained CNN

The overall aim of this study was to quantify how similarly CNNs process natural images and drawings of objects. This was accomplished by comparing their performance, their internal representations, and by comparing them to human behavior.

#### Similarity in terms of classification performance

**Figure 3.**
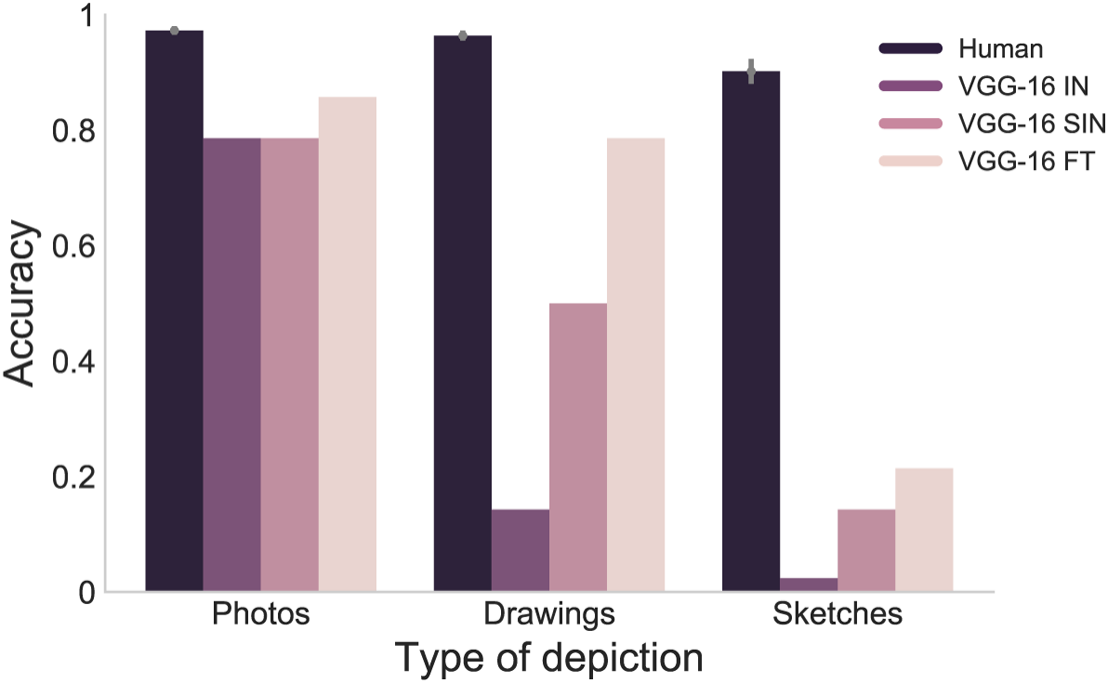
**Classification performance in variants of VGG-16 in comparison to humans.** Human accuracies measured in a separate online experiment revealed that humans performed very well and virtually identical on photos and drawings and slightly worse on sketches. The ImageNet-trained VGG-16 (VGG-16 IN) showed high performance on photos but a sharp drop in performance for drawings and sketches. VGG-16 trained on stylized ImageNet (VGG-16 SIN) performed better on drawings than VGG-16 IN but not on sketches or photos. VGG-16 fine-tuned on ImageNet-Sketch (VGG-16 FT) performed equally well on photos and drawings but still performed poorly on sketches.

As a first step, we sought to test how an ImageNet-trained CNN, specifically VGG-16 (Simonyan & Zisserman, 2015), generalizes to drawings and sketches in terms of classification performance. For this purpose, we passed the images through the network and compared predicted class labels with the actual object categories. We found that VGG-16 exhibited excellent classification accuracy for photos but very low accuracies for drawings and sketches (Figure 3). Statistical comparisons of the accuracies revealed that drawing performance was significantly lower than photo performance (*M*(Photos) = 0.79, *M*(Drawings) = 0.14, χ²(1) = 33.44, *p* < 0.001, false discovery rate [FDR]-corrected). Sketch performance was also significantly lower than photo performance (*M*(Sketches) = 0.02, χ²(1) = 47.25, *p* < 0.001, FDR-corrected) but there was no significant difference between the network's performance on drawings and sketches (χ²(1) = 0.96, *p* = 0.326, FDR-corrected).

To compare the network's performance to humans, we measured human classification accuracy on the same images in a separate online labeling experiment. Unlike VGG-16, humans performed very well and virtually identical on photos and drawings (*M*(Photos) = 0.97, *M*(Drawings) = 0.96, *t*(41) = −1.93, *p* = 0.030, two one-sided tests of equivalence) and showed only a slight but significant decrease in performance for sketches (*M*(Sketches) = 0.90, photos versus sketches - *t*(82) = 3.12, *p* = 0.008, FDR-corrected; drawings versus sketches - *t*(82) = 2.67, *p* = 0.014, FDR-corrected). The network performed significantly worse across all types of depiction as compared to humans (all *p* = 0.002, FDR-corrected, one-sided randomization test). These results demonstrate that VGG-16 pretrained on ImageNet fails to replicate human generalization to drawings and sketches in terms of classification performance.

#### Similarity in terms of internal representations

To compare VGG-16 not only in terms of performance but also regarding its internal representations of photos, drawings, and sketches, we used RSA (Kriegeskorte et al., 2008). For a given type of depiction, we calculated the pairwise dissimilarities (1 - Pearson *r*) for all pairs of object activation patterns and stored them in a RDM. This procedure yielded three RDMs (one for each type of depiction) for each of the layers in the network. Finally, we computed the Spearman rank correlation between the lower triangular part of RDMs of different types of depiction. This yielded three sets of comparisons: photo-to-drawing similarity, photo-to-sketch similarity, and drawing-to-sketch similarity. We assumed that if the network processed object images at different levels of visual abstraction similarly, this would be reflected in a similar representational format across types of depiction.

**Figure 4.**
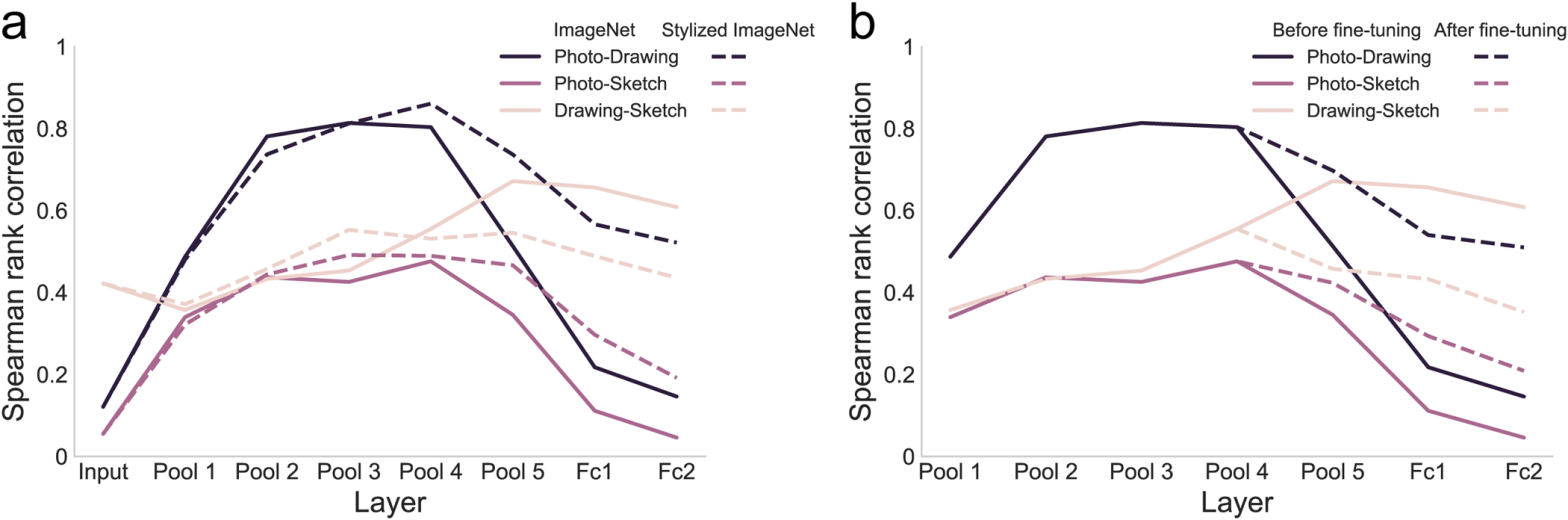
**Similarity in representational structure between types of depiction in variants of VGG-16.** (**A**) Spearman rank correlations between RDMs for the different types of depiction based on the CNN input (raw pixel values of preprocessed images) as well as activations in the ImageNet-trained VGG-16 (VGG-16 IN) and activations in the VGG-16 trained on stylized ImageNet (VGG-16 SIN). Based on the CNN input, we found a low similarity between photos and both drawings and sketches. The similarity between drawings and sketches, however, was higher. After passing the images through the network, we observed a high degree of representational similarity between photos and drawings and to a lesser extent also between photos and sketches in early and intermediate layers in both networks. In the later layers, these similarities between photos and abstracted types of depiction dropped sharply in VGG-16 IN, whereas in VGG-16 SIN this drop was attenuated. (**B**) Spearman rank correlations between RDMs for the different types of depiction in the ImageNet-trained VGG-16 before and after fine-tuning. After fine-tuning, the similarity in the representational format was increased for the photo-to-drawing and photo-to-sketch similarity, but reduced for the drawing-to-sketch similarity, indicating increased similarity in processing between photos and abstracted types of depiction in the network after fine-tuning.

First, as a baseline measure of similarity between types of depiction, we determined the similarity which is given only by the input to VGG-16. For this, we computed RDMs based on the raw pixel values obtained after preprocessing the images and before passing them through the network. Correlating the RDMs based on the raw pixel values between types of depiction, we found that the similarity between photos and drawings and between photos and sketches was low and not significantly different (*p* = 0.141, one-sided randomization test, FDR-corrected; Figure 4A). The correlation between drawings and sketches, however, was higher than the other two correlations (both *p* < 0.003, one-sided randomization test, FDR-corrected). This suggests that while the raw image structure is similar for drawings and sketches, it is very dissimilar for photos and drawings as well as for photos and sketches.

Comparing the degree of representational similarity between photos, drawings, and sketches based on the activations in VGG-16 and how it changes across layers, we found overall differences in RDM correlations across layers for all three comparisons (all *p* < 0.001, one-sided randomization test). We followed up on these overall differences with tests between pairs of layers for one type of depiction. For the photo-to-drawing similarity, we found a significant increase in correlation from pooling layer one to pooling layer four (*p* = 0.002, one-sided randomization test, FDR-corrected) and after that a significant drop in similarity from pooling layer four to fully connected layer two (*p* = 0.002, one-sided randomization test, FDR-corrected; see Figure 4A). While there were overall relatively lower correlation values for the photo-to-sketch correlation (all *p* < 0.003, one-sided randomization test, FDR-corrected), the shape of the results was similar, with an increase in similarity from pooling layer one to pooling layer four (*p* = 0.005, one-sided randomization test, FDR-corrected) and a drop in correlation for higher layers which approached zero in the penultimate layer (*p* = 0.003, one-sided randomization test, FDR-corrected). These results demonstrate a representational format which is shared between photos and drawings at intermediate layers and a weaker but stable similarity to sketches.

Interestingly, a different pattern was observed for the drawing-to-sketch similarity. Here, we found an increase in correlation from pooling layer one to pooling layer four (*p* = 0.003, one-sided randomization test, FDR-corrected) and no significant difference in correlation between pooling layer four and the fully connected layer two (*p* = 0.309, one-sided-randomization test, FDR-corrected).

Overall, the results reveal an increase in representational similarity across levels of visual abstraction that peaks in intermediate layers. This pattern of results diverged after pooling layer four, with photo representations becoming less similar to drawings and sketches, whereas drawing and sketch representations stay at a highly similar level.

#### Contribution of training and architecture to the similarity in representations between types of depiction

**Figure 5.**
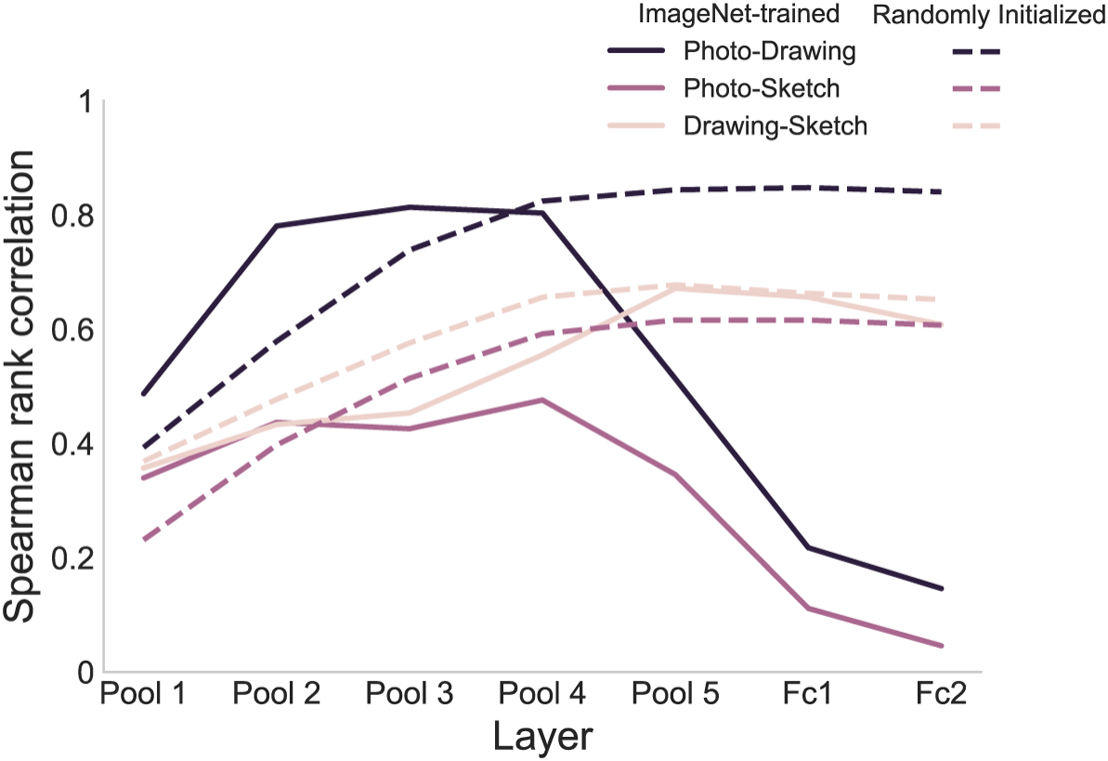
**Representational similarities between types of depiction in an ImageNet-trained and a randomly initialized instance of VGG-16.** The rise in representational similarities between photos and both drawings and sketches in early and intermediate layers was mirrored in the randomly initialized and ImageNet-trained variant of VGG-16. Yet, similarities between photos and abstracted types of depiction were stronger after training in these layers. In contrast, in the late layers, similarities between photos and both drawings and sketches remained high in the randomly initialized variant of VGG-16, whereas they dropped in the ImageNet-trained network. These results indicate that while the architecture explains part of the observed representational similarities between photos and both drawings and sketches, training influences these similarities both positively in early layers as well as negatively in late layers. Finally, for the drawing-to-sketch similarity, there were no significant differences between the randomly initialized and the ImageNet-trained VGG-16.

Furthermore, we aimed at disentangling the contribution of training and architecture to the observed representational similarities between types of depiction. To this end, we ran our stimuli through a variant of VGG-16 with randomly initialized weights and obtained RDMs for the different types of depictions across layers. Correlating these RDMs between types of depiction, we found that for all comparisons there were significant differences in correlations for all comparisons across layers (all *p* < 0.001, one-sided randomization test). Pairwise tests revealed an increase in correlation for all comparisons between pooling layer one and pooling layer four (all *p* < 0.003, one-sided randomization test, FDR-corrected; Figure 5). After pooling layer four, similarities either increased further for the photo-to-drawing comparison (*p* = 0.041, one-sided randomization test, FDR-corrected) or remained at a level that was not significantly different for the photo-to-sketch and drawing-to-sketch comparisons (all *p* > 0.318). Next, we directly compared the similarities from the randomly initialized VGG-16 and the ImageNet-trained variant. For the photo-to-drawing similarity, the similarities were either not significantly different or higher in VGG-16 IN in the early layers up to pooling layer four (pool 1: *p* = 0.263; pool 2: *p* = 0.005; pool 3: *p* = 0.033; and pool 4: p = 0.059, one-sided randomization test, FDR-corrected). This pattern reversed in the late layers from pooling layer five, with significantly lower similarities in VGG-16 IN than in the randomly initialized network (all *p* < 0.005). A similar pattern was found for the photo-to-sketch similarity; in early layers, there were no differences up to pooling layer four (all *p* > 0.123, one-sided randomization test, FDR-corrected), whereas, in late layers, similarities were lower for VGG-16 IN (all *p* = 0.006). Finally, for the drawing-to-sketch comparison, there were no significant differences in similarity between both networks (all *p* > 0.163). In sum, this suggests that at least a part of the representational similarities between photos and both drawings and sketches in early and intermediate layers can be accounted for by the architecture of the network. Yet, these results indicate that training improved the similarities even further in these layers. In contrast, the drop in representational similarities in late layers cannot be explained by the architecture alone and may be related to biases induced by the training of the network. Finally, the similarity between drawings and sketches appears to be unaffected by training.

#### Clustering of drawing and sketch representations

**Figure 6.**
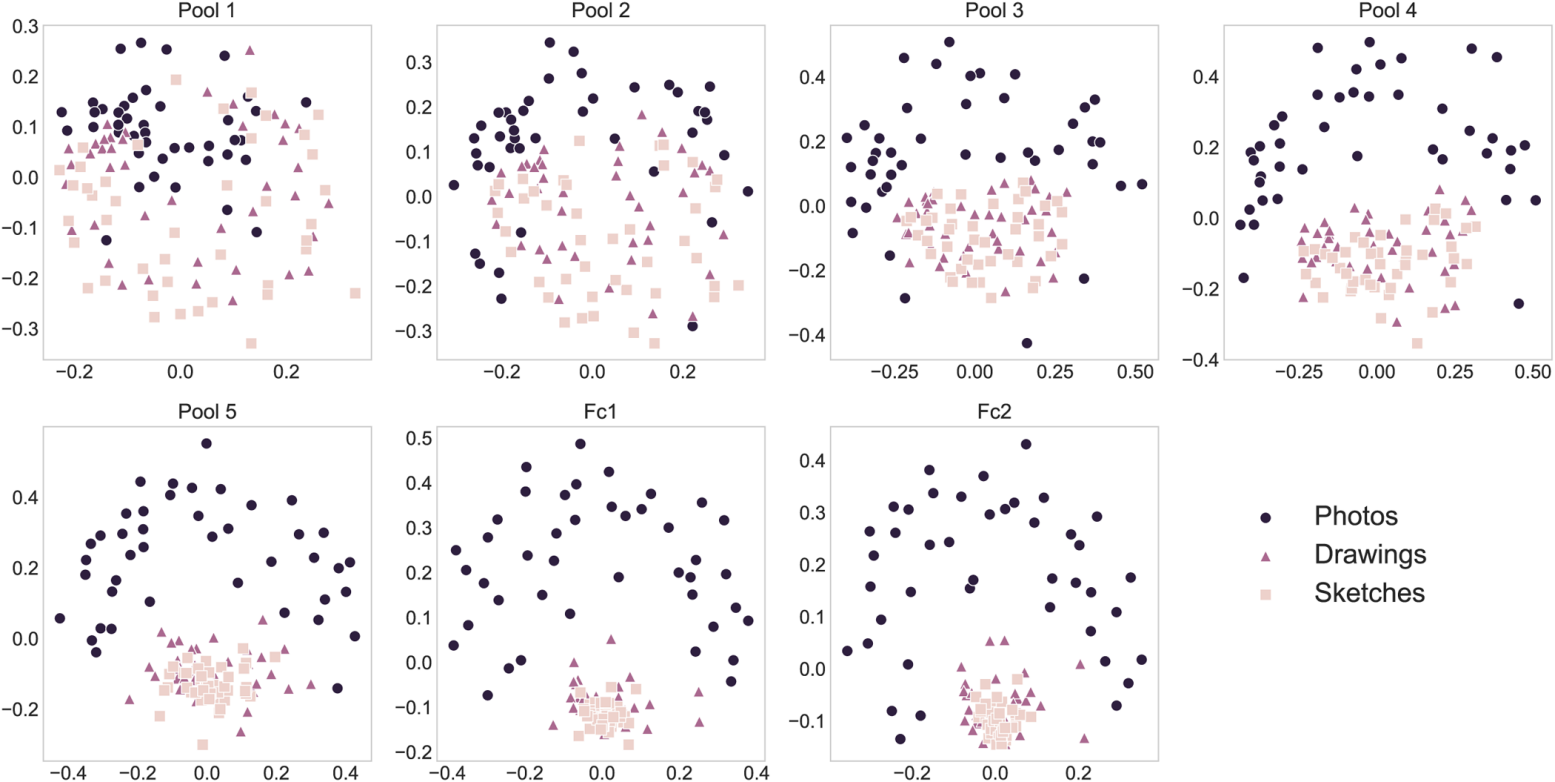
**Clustering of abstracted object representations in the ImageNet-trained VGG-16.** Multidimensional scaling plots of the super-RDMs reflecting all pairwise dissimilarities between stimulus representations in the stimulus set. Across layers, drawing and sketch representations increasingly clustered together, whereas photo representations remained well separated.

The sharp drop in representational similarity for the photo-to-drawing and photo-to-sketch comparison in the ImageNet-trained VGG-16 suggests a strong shift in the representational structure after pooling layer four. To illustrate the source of this drop, we visualized all representational similarities using multidimensional scaling. To this end, for a given layer, we first constructed a super-RDM composed of the pairwise distances between all pairs of stimuli in the set (see Appendix A2). Subsequently, we used metric multidimensional scaling to project the representational dissimilarities into a two-dimensional (2D) space. We then repeated this entire procedure for each layer of the selected layers of VGG-16. In these 2D projections, we found that the activations for all types of depiction appeared evenly distributed in the first two pooling layers. However, starting already at pooling layer three, activations for drawings and sketches started to cluster into one combined cluster, and the degree of clustering showed an increase up to the penultimate layer (Figure 6).

#### No collapse of representations for drawings and sketches

At first, this strong clustering might indicate a “representational collapse” of drawing and sketch representations toward specific points in the representational space. This might be expected, given the dramatic difference in image statistics between natural images the network had been trained on and drawings and sketches. A strong interpretation of this account would predict that all images of drawings and sketches are treated as largely equal by the network, possibly all leading to the same incorrect predicted category, and with differences in representations between images only reflecting meaningless residual noise. However, the fact that representational similarities between drawings and sketches remain high indicates that there is still some representational structure shared between these types of depiction. What is left open is to what extent these representations are idiosyncratic but similar, due to the similar appearance of drawings and sketches, or whether the representation still carries meaningful information, for example, about the high-level category of an object.

**Figure 7.**
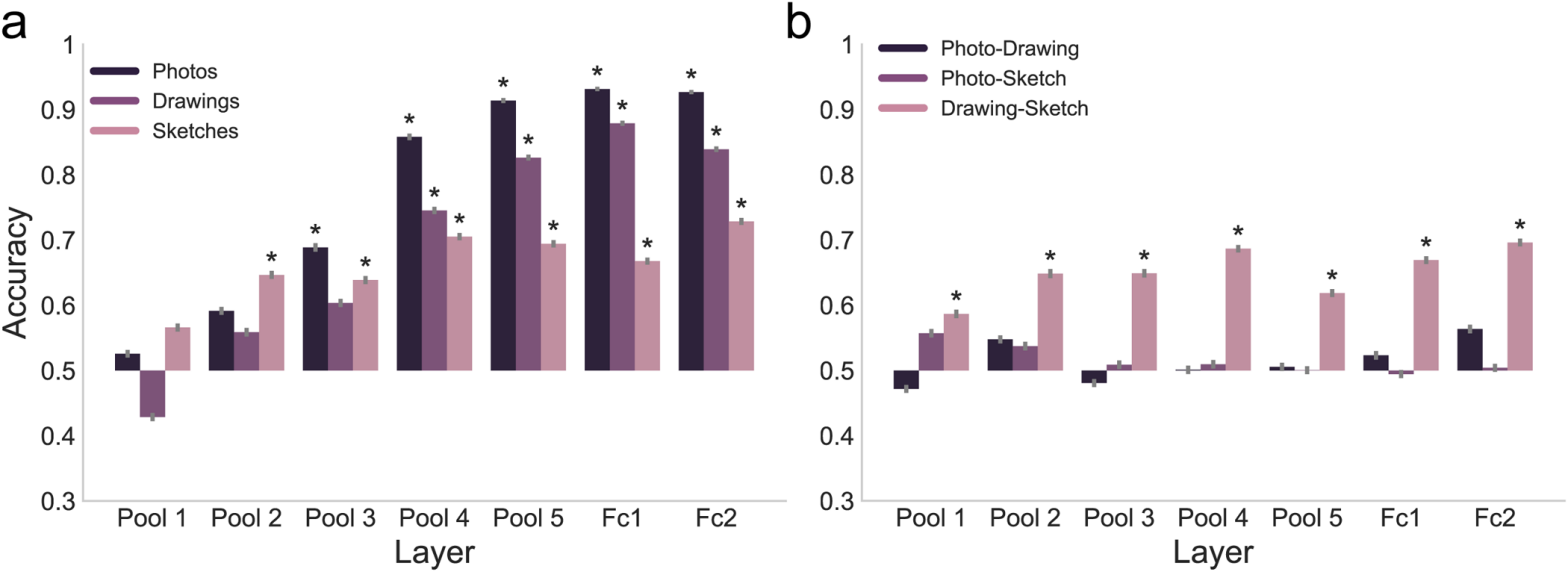
**Classification of high-level category information based on feature representations across layers in VGG-16.** (**A**) Classification accuracies in VGG-16 IN. Based on the features extracted from single layers in VGG-16 IN high-level category information (manmade/natural classification) could be decoded with above-chance accuracy even for drawings and sketches, particularly in the later layers. (**B**) Cross-classification accuracies in VGG-16 IN. Generalization of the classifier trained on one type of depiction (e.g. photos) to another type (e.g. drawings) showed above-chance accuracy only for the case where we trained on drawings and tested on sketches.

To test for the representational content of the drawing and sketch representations, we used a Support Vector Machine classification approach to predict an object's high-level category (manmade/natural) from the network's activation pattern, separately for each layer and type of depiction. For photos, we found significant above-chance classification accuracies from pooling layer three onward (all *p* < 0.011, one-sided randomization test, FDR-corrected). A similar effect was observed for drawings from pooling layer four (all *p* = 0.004, one-sided randomization test, FDR-corrected), and for sketches starting from pooling layer two (all *p* < 0.048, one-sided randomization test, FDR-corrected; Figure 7A). These results indicate that despite the strong clustering observed in the RDMs as visualized by the MDS plots, category-relevant information was retained in the activation patterns of drawings and sketches in the later layers. These results argue against a strong representational collapse (as described above) for drawings and sketches. Instead, they suggest a representational shift, indicating that the relative representational structure of objects is not destroyed and still allows meaningful readout of category information.

#### Generalizable high-level information for drawings and sketches

The classification results indicate that category information is still present in drawing and sketch representations but leaves open to what degree this information generalizes between types of depiction. To test this, we carried out cross-classification, training a Support Vector Machine classifier on the manmade/natural distinction using activation patterns from one type of depiction, and testing it on another type. We found significant above-chance cross-classification accuracies only for the case in which we trained on drawings and tested on sketches (all *p* < 0.041, one-sided randomization test, FDR-corrected), however, not for the cases where we trained on photos and tested on drawings or tested on sketches (all *p* > 0.372, one-sided randomization test, FDR-corrected; Figure 7B). This suggests that the information contained in the representation only generalizes between drawings and sketches but not between photos and any of the other types of depiction. This is consistent with the idea that the representations of drawings and sketches do not collapse but are shifted and thus retain some meaningful information, which, however, exhibits a different format than the information for photos.

#### Fit to human behavior

The classification of a single high-level object category might be seen as insufficient evidence of intact representations. For that reason, we obtained human behavioral similarity measurements to quantify how the objects in our stimulus set are represented in humans (see Appendix A4 for visualization) and to compare these human representations to the network's representations. Assuming that there was a collapse in the representations for drawings and sketches that increases across layers, we would expect to see a drop in representational similarity between VGG-16 and human behavior, both for drawings and sketches, specifically in the late layers. Alternatively, in the case of a shift of representations, we would expect smaller representational similarities for drawings and sketches than for photos but still significant representational similarities for these types of depiction in later layers. This would in turn indicate that there is some meaningful structure preserved in the representation for drawings and sketches, which is similar to human behavior. To examine this, we correlated the RDMs that we obtained for the layers of VGG-16 separately for every layer and type of depiction with the corresponding RDM that we obtained from human similarity judgments.

**Figure 8.**
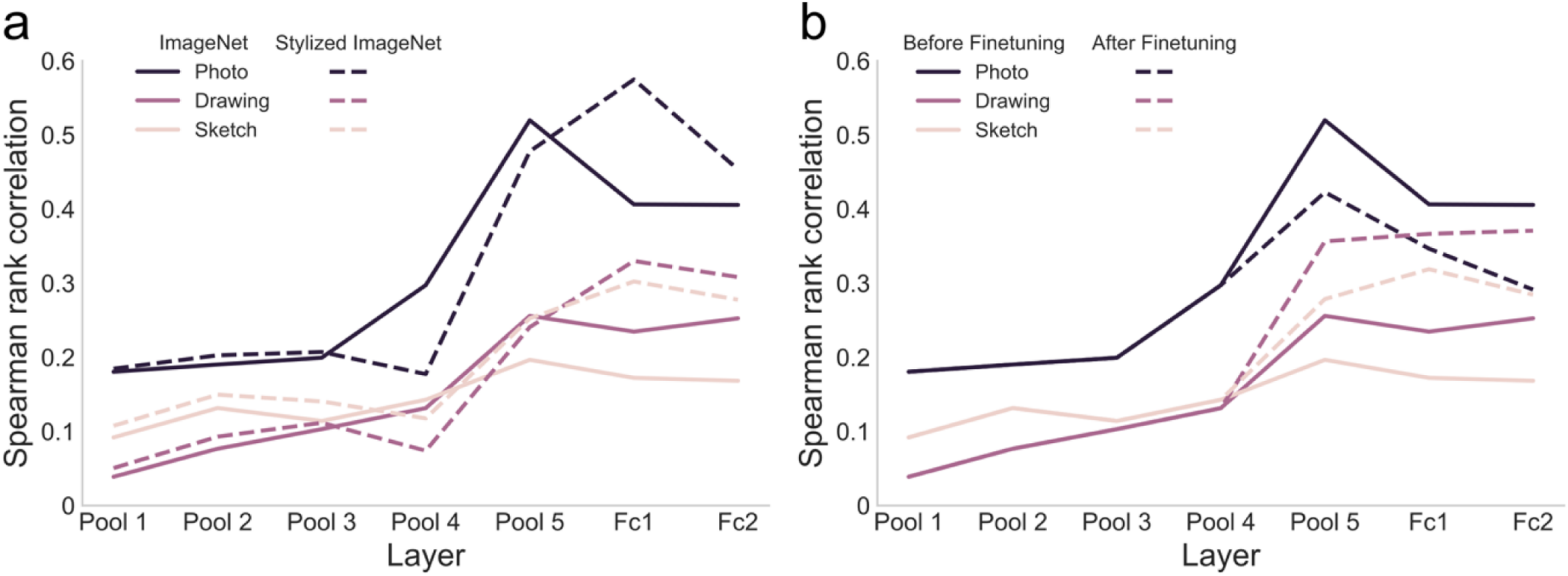
**Fit to human behavior in variants of VGG-16.** (**A**) Spearman rank correlations between the RDMs obtained from the ImageNet-trained VGG-16 (VGG-16 IN) and the VGG-16 trained on stylized ImageNet (VGG-16 SIN) for every layer in the networks and the RDMs obtained in an online behavioral experiment, for each type of depiction separately. We observed the best fit to human behavior in VGG-16 IN for the representations of photos. Although lower, we found a significant fit to human behavior for the drawing and sketch representations. In VGG-16 SIN the fit to human behavior for drawings and sketches mostly improved in comparison to VGG-16 IN. (**B**) Spearman rank correlations between the RDMs obtained from the ImageNet-trained VGG-16 (VGG-16 IN) and the fine-tuned VGG-16 and the RDMs obtained from human behavior. Fine-tuning increased the similarity of drawing and sketch representations to representations in human behavior in all fine-tuned layers. For photos, however, the similarity to human behavior was decreased after fine-tuning.

Perhaps not surprisingly, we found the highest fit of network representations to human behavior for photos (Figure 8A), showing an increase in RDM correlation up to pooling layer five, after which the correlation dropped slightly in the penultimate layer of the network. We observed a similar pattern for the drawings and sketches with an increase in RDM correlation up to pooling layer five, after which the correlation stabilized in the fully connected layers. Statistical tests confirmed that there were significant correlations in all layers for photos and sketches (all *p* < 0.011, one-sided Mantel test, FDR-corrected) and starting at pooling layer three for drawings (all *p* < 0.036, one-sided Mantel test, FDR-corrected).

Comparing the RDM correlations against each other revealed that the RDM correlation for photos was significantly higher than for drawings in all layers (all *p* < 0.005, one-sided randomization test, FDR-corrected) and for sketches (all *p* < 0.039) in all layers but pooling layer two (*p* = 0.103). The correlations for drawings and sketches were only significantly different in the fully connected layers (both *p* < 0.045) but not in all the other layers (all *p* > 0.079).

In conclusion, we found the best fit of network representations to human behavior for representations of photos. Importantly, however, we found a significant fit to human behavior for drawings and sketches, which increased across layers and remained high until the last layer. This is in line with the idea of a shift in representations of abstracted object images. While the representations are shifted, they are not completely distorted and retain category-relevant information of the objects and show similarity to human representations.

Taken together, the results of experiment 1 suggest fairly general object representations in intermediate layers, which show generalization across different levels of visual abstraction. Yet, in the process of linking these representations to object categories, they are presumably biased toward the object features in natural images, leading to dissimilar representations in later layers and low network classification accuracies for drawings and sketches. This would indicate that the issue of incorrect classification is not an issue of representation as such, but more an issue of read-out into object classification.

### Experiment 2: Generalization to drawings in a shape-biased CNN

In experiment 1, we found that the ImageNet-trained VGG-16 categorizes drawings and sketches poorly. However, in terms of its internal representations, there seems to be considerable similarity between photos and drawings, and to some extent between photos and sketches. Despite this similarity in intermediate layers, as image processing approached the classification stage, the network represented drawings and sketches very differently to photos. Yet, the CNN representations for drawings and sketches retained category-relevant information about the objects and showed similar representations to those derived from human similarity judgements. While these results highlight the representational nature of object drawings and sketches in VGG-16, they leave open the critical question of why the representations shift in later layers, which seems to cause misclassifications of the network.

One reason for the dissimilar representation of natural images and abstracted images in the late stages of processing and the poor performance for abstracted images might be that ImageNet-trained CNNs rely heavily on local texture information for their classifications (Baker, Lu, Erlikhman, & Kellman, 2018; Geirhos et al., 2019). Since texture information is strongly altered in both drawings and sketches, a reliance on texture information for the classification might favor a dissimilar representation of photos and abstracted images. In order to reduce the bias for texture in CNNs, Geirhos et al. (2019) introduced a new way of training CNNs, forcing them to rely more on shape information than texture. By training CNNs on a stylized version of the ImageNet dataset (Gatys et al., 2016), they revealed a preference of shape over texture in the classification behavior of the network. Assuming that, in experiment 1, texture bias indeed contributed to the mismatch between the representation of photos and abstracted object images in later layers, this leads to two predictions. First, we would expect that for a CNN trained on stylized images, the drop in representational similarity between photos and abstracted types of depiction in later layers is attenuated, because they now share more similar overall shape information in the network's representation. Second, we would expect improved classification performance on abstracted object images. To test the role of the texture bias in the representation of drawings in CNNs, we analyzed the processing for VGG-16 which was trained on stylized ImageNet analogous to experiment 1.

#### Better performance on drawings in a shape-biased CNN

Again, as a first step we focused on the performance of the network in terms of predicting the object category from the images, separately for photos, drawings, and sketches. We found that the network trained on stylized ImageNet (VGG-16 SIN) compared to the ImageNet-trained variant (VGG-16 IN), performed similarly well on photos (*M*(Photos) = 0.79, χ²(1) = 0.01, *p* = 0.913, FDR-corrected), however, showed much improved performance on drawings (*M*(Drawings) = 0.50, χ²(1) = 10.01, *p* = 0.005, FDR-corrected), with performance on sketches remaining poor (*M*(Sketches) = 0.14, χ²(1) = 0.96, *p* = 0.489, FDR-corrected; see Figure 3). Despite these improvements, performance in VGG-16 SIN on drawings was still worse than on photos (χ²(1) = 6.30, *p* = 0.121, FDR-corrected) and significantly worse on sketches than on drawings (χ²(1) = 10.01, *p* = 0.002, FDR-corrected). Similar to experiment 1, the direct comparison of VGG-16 SIN performance to humans revealed that the network performed significantly worse in all types of depiction than humans (all *p* < 0.001, one-sided randomization test, FDR-corrected).

In conclusion, VGG-16 SIN performed better on drawings than VGG-16 IN, however, the network still performed worse on drawings than on photos and did not perform better on sketches than VGG-16 IN.

#### Attenuated drop in representational similarity across levels of visual abstraction

Next, we quantified the similarity in processing between types of depiction in VGG-16 SIN in terms of the network's internal representations analogous to experiment 1. We hypothesized that we would see a particular attenuation of the drop in representational similarity toward the late layers compared to what we observed in experiment 1.

In comparison to VGG-16 IN, photo-to-drawing correlations in the early layers in VGG-16 SIN were either not statistically different from the ones in VGG-16 IN (pooling layer 1: *p* = 1; and pooling layer 3: *p* = 1, one-sided randomization test, FDR-corrected) or higher in VGG-16 IN (pooling layer 2: *p* = 0.004; see Figure 4A). In contrast, starting in pooling layer four, all photo-to-drawing correlations were higher in VGG-16 SIN (all *p* < 0.004, one-sided randomization test, FDR-corrected), reflecting indeed an attenuated drop in representational similarity between photos and drawings in VGG-16 SIN as compared to VGG-16 IN. A very similar pattern was found for the photo-to-sketch correlation. For early and intermediate layers up to pooling layer four, the only difference in similarity was found in pooling layer three, with higher values in VGG-16 SIN (*p* = 0.0121; all other *p* > 0.548, one-sided randomization test, FDR-corrected). For the later layers starting from pooling layer five, we found consistently higher values for VGG-16 SIN (all *p* = 0.009). Finally, for the drawing-to-sketch comparison, we observed higher values in VGG-16 SIN only in pooling layer three (*p* = 0.018, one-sided randomization test, FDR-corrected) but no significant differences in any of the other layers (all *p* > 0.105).

To summarize, while representational similarities between natural images and abstracted types of depiction in early and intermediate layers were mostly similar or lower in VGG-16 SIN compared to VGG-16 IN, in the later layers of the network we found consistently higher values in VGG-16 SIN. For the drawing-to-sketch similarity, we found overall mostly similar correlations in VGG-16 SIN and VGG-16 IN. Taken together, these results suggest a specific role of texture bias in the similarity in processing across levels of visual abstraction in late layers of VGG-16 but less so in the early and intermediate layers.

#### Increased similarity of drawing and sketch representations to human behavior in VGG-16 SIN

Given the improvements in classification performance and representational similarity between photos and both drawings and sketches in the network trained on stylized ImageNet, it could be expected that representations for drawings and sketches now also more closely resemble representations in human behavior. To test this, we correlated the RDMs derived from VGG-16 SIN for the three types of depiction with the corresponding RDMs obtained from human behavior and compared the correlations to those in VGG-16 IN. We found better fits to human behavior for photos in VGG-16 IN in pooling layers four and five (both *p* < 0.012, one-sided randomization test, FDR-corrected) but better fits in VGG-16 SIN in fully connected layer one (*p* = 0.009), with no significant differences in all other layers (all *p* > 0.077; see Figure 8A). For drawings, we found higher values in VGG-16 SIN in pooling layers one and two and fully connected layer one (all *p* < 0.014, one-sided randomization test, FDR-corrected) and higher values in VGG-16 IN in pooling layer four (*p* = 0.006). Finally, for sketches, we found differences in the fit to human behavior between the networks, with a better fit in VGG-16 SIN in all layers except for pooling layer four (pool 4: *p* = 0.075; all other *p* < 0.030, one-sided randomization test, FDR-corrected). In short, we found differences in fit to human behavior between VGG-16 IN and SIN for all types of depiction, with overall mostly higher fits in VGG-16 SIN for the abstracted types of depiction. Taken together, this suggests that the improved generalization in terms of performance and representations to abstracted types of depiction in VGG-16 SIN compared to VGG-16 IN is also reflected in its fit to human behavior.

### Experiment 3: Generalization through transfer learning

Reducing texture bias in VGG-16 improved the similarity in processing of object images across levels of visual abstraction particularly regarding the representational structure in late layers but mostly did not affect intermediate and early layers. This implies that the features in early and intermediate layers in the ImageNet-trained VGG-16 might already be general enough to support recognition of abstracted drawings. To directly test this, we used transfer learning, from the task of classifying natural object images to the task of classifying drawings and sketches. For this purpose, we fine-tuned VGG-16 pretrained on ImageNet on the ImageNet-Sketch dataset (Wang et al., 2019). We used representational similarities between the types of depiction as an index for the generality of representations in a given layer and based our selection of layers for transfer learning on that (Dwivedi & Roig, 2019). Hence, for training the network, we froze the connection weights of the network up to pooling layer four and only adapted the weights of the layers after pooling layer four, which includes the last convolutional block, the last pooling layer, and all fully connected layers.

#### Restored performance on drawings after fine-tuning

After fine-tuning, we found high and comparable performance of the network (VGG-16 FT) for photos and drawings (*M*(Photos) = 0.86, *M*(Drawings)=0.79, χ²(1) = 0.30, *p* = 0.584, FDR-corrected) but still lower and relatively poor performance for sketches (*M*(Sketches) = 0.21, photos versus sketches – χ²(1) = 33.44, *p* < 0.001, FDR-corrected; drawings versus sketches – χ²(1) = 26.30, *p* < 0.001, FDR-corrected; see Figure 3). When comparing VGG-16 before and after fine-tuning, we found no statistically significant difference between networks on photos (χ²(1) = 0.30, *p* = 0.585, FDR-corrected). Further, drawing performance was significantly higher in VGG-16 FT than in VGG-16 IN (χ²(1) = 33.44, *p* < 0.001, FDR-corrected) but sketch performance was not (χ²(1) = 2.68, *p* = 0.153, FDR-corrected). In direct comparison with human performance, we found that VGG-16 FT performed worse than humans on every type of depiction (all *p* < 0.001, one-sided randomization test, FDR-corrected), mirroring the results of experiments 1 and 2. Taken together, the fine-tuned network showed largely restored performance for drawings, yet, performance on sketches remained poor.

#### Increased representational similarity across levels of visual abstraction after fine-tuning

Since we explicitly fine-tuned the later layers on abstracted object images, we expected to see an attenuated decrease in representational similarity between photos and abstracted types of depiction toward the output layer as compared to VGG-16 IN in experiment 1, similar to what we observed for VGG-16 SIN in experiment 2. Indeed, after fine-tuning, the network showed higher photo-to-drawing and photo-to-sketch similarity compared to VGG-16 IN across all fine-tuned layers (all *p* < 0.049, one-sided randomization test, FDR-corrected) whereas drawing-to-sketch similarity was decreased (all *p* < 0.002; see Figure 4B).

In comparison to VGG-16 SIN, there were no significant differences in both the photo-to-drawing or photo-to-sketch similarity in any of the fine-tuned layers (all *p* > 0.264, one-sided randomization test, FDR-corrected). For the drawing-to-sketch correlation, there were significantly higher values in VGG-16 SIN only in pooling layer five (*p* = 0.017, one-sided randomization test, FDR-corrected) but not in fully connected layers one and two (both *p* = 0.209).

Together, this demonstrates that the drop in representational similarity between photos and both drawings and sketches was attenuated after fine-tuning. This attenuation was comparable to the one observed in VGG-16 SIN in experiment 2.

#### Improved fit to human behavior for drawings and sketches after fine-tuning

The fine-tuned network showed restored classification performance on drawings, and the drop in representational similarity between photos and abstracted types of depiction was attenuated. In order to test if fine-tuning also led to representations that resembled representations in human behavior more closely than before fine-tuning, we correlated the RDMs from the fine-tuned layers with the RDMs obtained from human behavior and compared this with the fit to human behavior in VGG-16 before fine-tuning. We found that after fine-tuning, the fit to human behavior was worse for photos but improved for both drawings and sketches in all fine-tuned layers (all *p* < 0.007, one-sided randomization test, FDR-corrected; see Figure 8B).

We conclude that the fine-tuning procedure improved the generalization to drawings regarding the classification performance of the network, the representational similarity across levels of visual abstraction, and the similarity to representations in human behavior for drawings and sketches. This suggests that given the right mapping between early and intermediate features learned on natural images and the classification layer, the network is able to similarly process photos and drawings. The performance for sketches, however, remained poor indicating that different recognition strategies might be required in the networks for high levels of visual abstraction.

## Discussion

The main objective of this study was to investigate to what extent generalization to abstracted object images is an emergent property of CNNs trained on natural images and how these generalization capabilities compare to those found in humans. To this end, we analyzed both the classification performance and the representational similarities across different levels of visual abstraction in VGG-16 and compared them to humans. In experiment 1, we showed that in the ImageNet-trained VGG-16, natural and abstracted object images evoke highly similar representations, with representational similarities peaking in intermediate layers. These similarities, however, dropped off sharply in later layers of the network, culminating in poor performance on drawings and sketches. In contrast, human behavioral performance was highly accurate and comparable across types of depiction. A similar rise in representational similarities between types of depiction as in the ImageNet-trained VGG-16 could be observed in a randomly initialized variant of VGG-16 suggesting that part of these similarities can be attributed to the architecture of the network. However, similarities between photos and drawings were improved in early and intermediate layers after training and the drop in later layers was not present in the randomly initialized network, in turn indicating that training affects the similarities in the network. Moreover, we observed that representations of drawings and sketches in the ImageNet-trained VGG-16 clustered together with increasing degrees across layers, whereas photo representations remained well separated, suggesting either a collapse or a shift in drawing and sketch representations. Two findings supported the idea of a representational shift: the presence of high-level category information in the representation for drawings and sketches, and their representational similarities to human behavior. Together, the results in experiment 1 suggest that photos, drawings, and sketches share strong similarities in processing up to intermediate layers in the network, which disappear with increasing proximity to the output layer, ultimately resulting in poor generalization to drawings and sketches. In experiment 2, we identified the degree to which a proposed texture bias in ImageNet-trained CNNs (Geirhos et al., 2019; Hermann et al., 2020) contributed to the drop in representational similarity and the poor generalization to drawings and sketches. We demonstrate that reducing texture bias in the network attenuated the drop off in representational similarity between types of depiction specifically in later layers. This, in turn, suggests that representations in early and intermediate layers of the ImageNet-trained VGG-16 are already general enough to serve as the foundation for the recognition of drawings. In experiment 3, we provide direct evidence for this notion by showing that increased performance for drawings was achieved by fine-tuning only the later layers in the network on a set of drawings while keeping the features in early and intermediate layers learned on ImageNet intact. This demonstrates that large parts of the ImageNet-trained VGG-16 are general enough to support recognition of drawings, yet, not for more abstract sketches. In the later stages of the network, however, the processing is presumably biased toward the image features and texture the network was trained on. This apparent bias favors a dissimilar representation for natural and abstracted object images and prevents the network from accurately classifying drawings and sketches.

Our findings reconcile the demonstrations of poor performance of CNNs on drawings (Ballester & Araujo, 2016; Evans et al., 2021; Wang et al., 2019) with evidence for generalizable representations for natural images and drawings (Fan et al., 2018). Our results furthermore suggest that processing of object images across levels of visual abstraction remains domain-general up to the penultimate pooling layer, after which a distinct representational format emerges that is more biased toward the domain of natural images and leads to incorrect classifications on drawings and sketches. Our observations in the different parts of processing in the networks are in line with distinct bodies of research on the processing in CNNs. While several studies find evidence for abstract shape representations and brain-like shape sensitivity in CNNs (Kalfas et al., 2018; Kubilius et al., 2016; Pospisil, Pasupathy, & Bair, 2018) others point out the specificity of these networks for the domain of their training data, in particular regarding their classification decisions (Geirhos et al., 2019; Geirhos, Temme, Rauber, Schütt, Bethge, & Wichmann, 2018; Goodfellow, Shlens, & Szegedy, 2015; Hermann et al., 2020). We propose that both lines of observations might be explained with a dynamically changing representational format that is differentially influenced by shape and texture information depending on the processing stage in the network. A recent study provides further evidence for this notion by showing that the number of shape and texture encoding units varies across processing stages in CNNs (Islam, Kowal, Esser, Jia, Ommer, Derpanis et al., 2021). However, shape and texture are clearly not the only features that influence representations in CNNs. Hence, further research investigating similarities and differences in the representational structure for different types of stimuli across processing stages is needed to provide a better understanding of the nature of representations in CNNs.

While we found high representational similarities between types of depiction in the early and intermediate layers in the ImageNet-trained variant of VGG-16, we found a similar increase in similarities in a randomly initialized instance of VGG-16. In addition, representational similarities between photos and both drawings and sketches remained high in the randomly initialized VGG-16 while they dropped sharply in the ImageNet-trained variant. On the one hand, this indicates that feedforward processing through a hierarchy of convolutional layers inherently leads to increased similarity between representations of photos, drawings, and sketches even when the convolutional filters are purely random. This is in line with recent findings proposing that random convolutions preserve local shape while discarding texture and that random convolutions can be used as a data augmentation technique to improve out-of-domain generalization to, for example, drawing images (Xu, Liu, Yang, Raffel, & Niethammer, 2021). On the other hand, our findings suggest a dual role of training the network for the representational similarity between types of depiction. First, training led to an increase in representational similarities in early and intermediate layers, indicating that learned features support the representational similarities between types of depiction. These features may include local edge features in early convolutional layers (Krizhevsky et al., 2012) or curvature features in intermediate layers (Cammarata, Goh, Carter, Schubert, Petrov, & Olah, 2020). In contrast to these results in early layers, training decreased the representational similarities in later layers, likely reflecting the bias of the network for the statistics of natural images, which is found in the photos but neither the drawings nor the sketches. Together, these observations imply that while the architecture of the network provides the basis for generalization to drawings, training in the network both contributes to the generalization in early layers and prevents it in later layers.

Another possible explanation for the limited similarities between natural and abstracted object images is related to the specifics of the chosen neural network architecture. One such choice is the type of layer in the network. While the decline in representational similarity was found already in the final pooling layer, the presence of fully connected layers might have contributed to the drop in performance. However, results of a similar analysis using a network consisting only of convolutional layers (vNet; Mehrer, Spoerer, Jones, Kriegeskorte, & Kietzmann, 2021) continued to yield a drop in classification performance for drawings and sketches, paired with decreases in representational similarities across levels of visual abstraction, albeit weaker than those found for VGG-16 (Appendix text, Appendix A5). This demonstrates that limitations in generalization to drawings in experiment 1 cannot be explained solely by the presence of fully connected layers. Future studies need to identify the degree to which the generalization performance can be maintained by changes in network architecture alone or whether alternative training regimes become necessary.

The fact that we found similarities between drawing and sketch representations in VGG-16 and representations in human behavior indicates that despite not being able to categorize drawings and sketches correctly, the network still captures meaningful information in its representation for drawings and sketches. However, it should be noted that the triplet task with which we measured human behavioral similarities allows participants to use information that CNNs might not capture in their representations. For example, humans might use information about the context in which objects typically appear to make inferences about which objects are more or less similar to each other. While there is evidence that CNNs represent contextual information, the way this information is represented in the human brain and CNNs seems to be markedly different (Bracci, Mraz, Zeman, Leys, & de Beeck, 2021). Nonetheless, the demonstration that there is similarity in representations between humans and CNNs, despite possible differences in availability of information, supports the conclusion that the network retains meaningful information about drawings and sketches despite shifts in the representation. It remains an open question to what extent such similarities can be accounted for by low-level perceptual or high-level contextual information, which invites further investigation.

We demonstrated that similarity in processing objects across visual abstractions is, in part, an emergent property in CNNs trained on natural images. Yet, despite attenuating texture bias and fine-tuning on drawings, the performance on very abstract sketch images remained poor. We propose two possible explanations for this discrepancy. First, ImageNet-Sketch (Wang et al., 2019) mostly contains highly detailed drawings, which more closely resemble the level of visual abstraction of the drawings in our stimulus set compared to the one of sketches. This might make ImageNet-Sketch a more suitable training dataset for drawings in our stimulus set than for sketches. Alternatively, the recognition of highly abstracted and simplified object images might require incorporating different mechanisms in CNNs than the ones used for classifying natural images and drawings. A possible mechanism for such robust recognition abilities might include some form of global shape processing. This ability is limited in predominant CNN architectures (Baker et al., 2018; Baker, Erlikhman, & Kellman, 2020) and may involve computations including recurrent segmentation and grouping which are not performed by canonical CNNs (Doerig, Schmittwilken, Sayim, Manassi, & Herzog, 2020). Investigating how this challenge is solved by networks with different architectures and processing mechanisms than current CNNs might elucidate the mechanisms that enable broad generalization abilities, including those for abstract sketches.

While the generalization capabilities in human recognition are still out of reach for networks with a feedforward architecture and supervised learning regime (Geirhos et al., 2019; Geirhos, Janssen, et al., 2018; Geirhos, Temme, et al., 2018), developing models that more closely match the human brain in terms of architecture (Evans et al., 2021; Kietzmann, Spoerer, Sörensen, Cichy, Hauk, & Kriegeskorte, 2019) and learning rules (Zhuang, Yan, Nayebi, Schrimpf, Frank, DiCarlo, et al., 2021) offer new perspectives for meeting this goal. Such models have yielded important insight in how the brain solves the general problem of object recognition and show improved generalization in some cases (Geirhos, Narayanappa, Mitzkus, Bethge, Wichmann, & Brendel, 2020; Spoerer, McClure, & Kriegeskorte, 2017) but are still outmatched by humans (Geirhos, Meding, & Wichmann, 2020; Geirhos, Narayanappa, et al., 2020). Only recently, a new class of models based on the concept of natural language supervision has been introduced, which show intriguingly robust performance across domains and across visual abstractions (Radford, Kim, Hallacy, Ramesh, Goh, Agarwal, et al., 2020). It is an open and exciting question to what extent these models and their training approach resemble principles of natural intelligence, which might ultimately provide new explanations for the remarkable generalization in human vision.

## Conclusions

Our results show that a CNN trained on natural images represents photos and drawings of objects in intermediate layers highly similarly while simultaneously exhibiting low similarities between types of depiction in late layers and performing poorly on drawings and sketches. The texture bias present in CNNs (Geirhos et al., 2019; Hermann et al., 2020) contributes to the low representational similarities between types of depiction in later layers and the network's reduced performance for drawings. After fine-tuning these late layers on a set of object drawings, the network showed high performance on photos and drawings and increased representational similarities between types of depiction in late layers. In conclusion, these results contribute to the understanding of generalization to drawings in CNNs, by revealing high similarities in processing of natural images and abstracted object images in the network and providing explanations for the link between these similarities and the limitations in performance for abstracted object images.

## Supplementary Material

Supplement 1

Supplement 2

Supplement 3

Supplement 4

Supplement 5

Supplement 6
