# Supplementary material for: From photos to sketches - how humans and deep neural networks process objects across different levels of visual abstraction
Source: J Vis. 2022 Feb 7;22(2):4. doi: 10.1167/jov.22.2.4 (PMC8822363; doi:10.1167/jov.22.2.4)
Supplement: Supplement 1 [file jovi-22-2-4_s001.pdf]

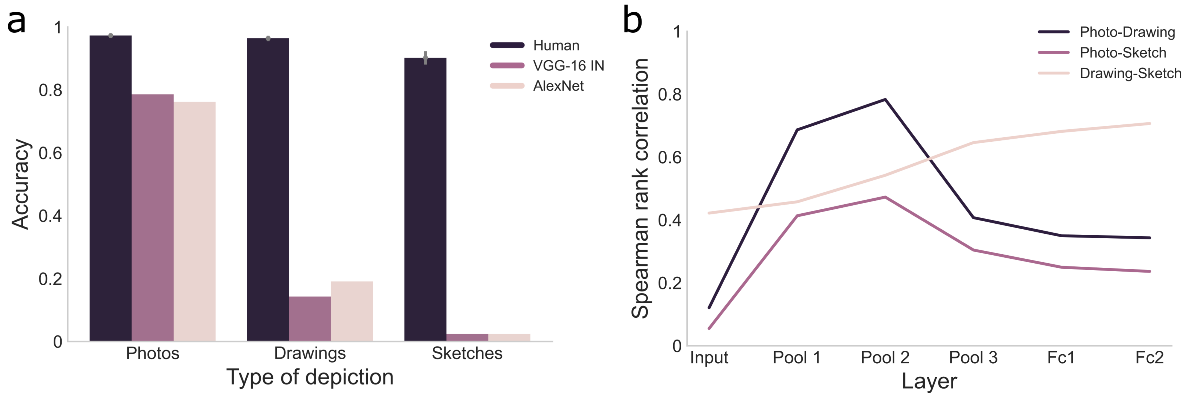

A1. Similarity in processing across levels of visual abstraction in AlexNet. a) Top-1 accuracies across types of depiction for humans, VGG16-IN and AlexNet. In comparison to humans, both AlexNet and VGG-16 IN perform poorly on drawings and sketches while showing decent and comparable performance on photos. **b)** Representational similarities between types of depiction based on CNN input and activations in AlexNet. Representational similarities between photos and both drawings and sketches first increased in pooling layer 1 and 2 in comparison to the similarity given by the CNN input. After that, for both comparisons the similarities dropped in pooling layer 3 and the fully connected layers. In contrast, the drawing-to-sketch similarities steadily increased across layers.
