# Supplementary material for: From photos to sketches - how humans and deep neural networks process objects across different levels of visual abstraction
Source: J Vis. 2022 Feb 7;22(2):4. doi: 10.1167/jov.22.2.4 (PMC8822363; doi:10.1167/jov.22.2.4)
Supplement: Supplement 2 [file jovi-22-2-4_s002.pdf]

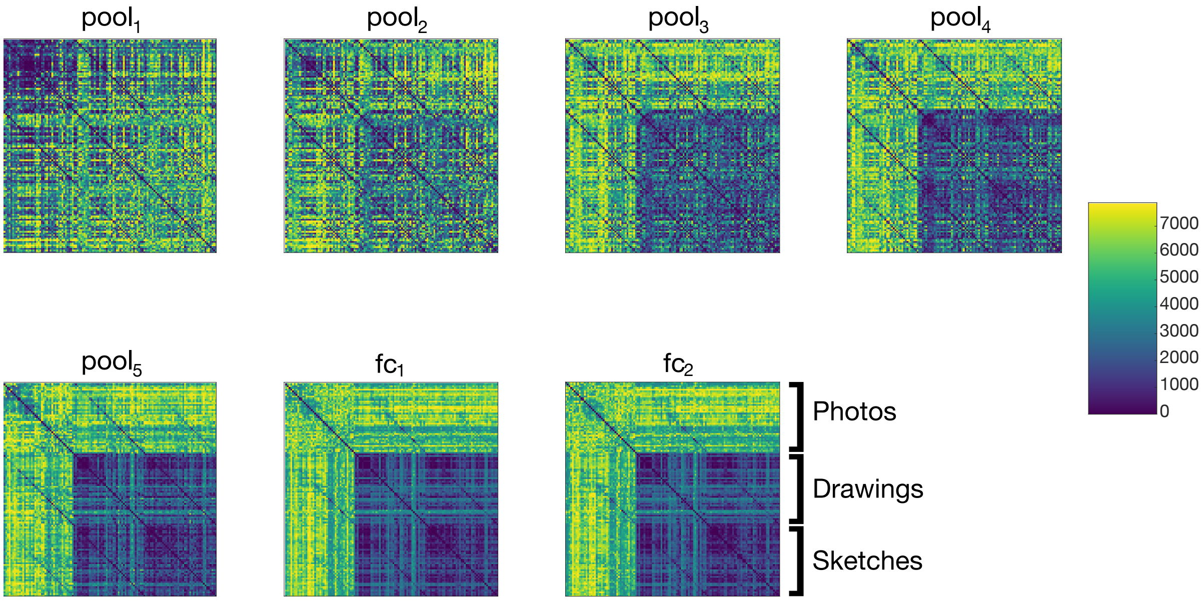

A2. Representational dissimilarity matrices containing all pairwise distances between all stimuli in the set across types of depiction (super-RDMs) for VGG-16. Similar to the MDS visualization starting already in pooling layer 3 the distances for drawings and sketches within and between the two types of depiction became visibly smaller than the distances for within photos and between photos and the abstracted types of depiction, which increased up to the last fully connected layer. For visualization purposes all distances in one RDM were ranked and sorted according to their superordinate category (manmade/natural) within one type of depiction.
