# Supplementary material for: From photos to sketches - how humans and deep neural networks process objects across different levels of visual abstraction
Source: J Vis. 2022 Feb 7;22(2):4. doi: 10.1167/jov.22.2.4 (PMC8822363; doi:10.1167/jov.22.2.4)
Supplement: Supplement 3 [file jovi-22-2-4_s003.pdf]

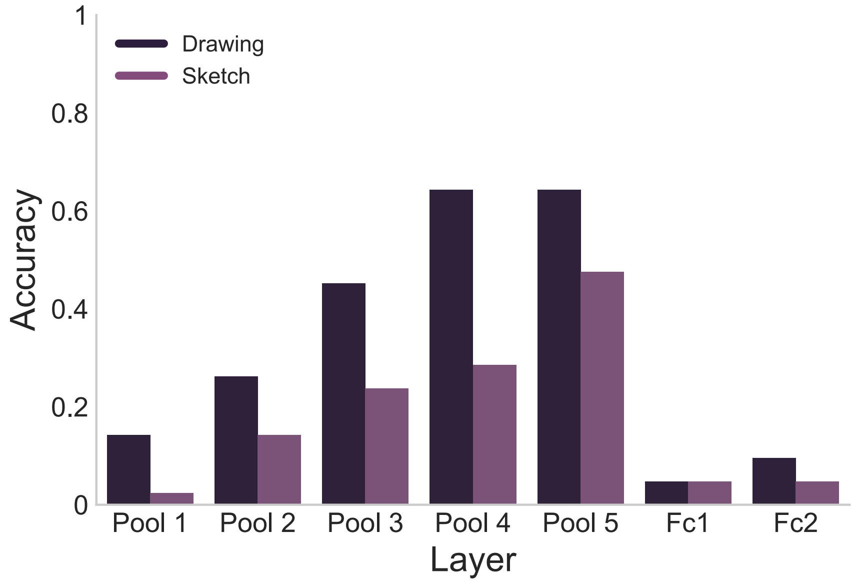

A3. Classification of category-specific information for drawings and sketches across layers in VGG-16. Category-information could be extracted with increasing accuracies across the pooling layers from activations in VGG-16 for both drawings and sketches. Yet, category-specific information was largely lost in the fully connected layers for both types of depiction.
