# Supplementary material for: From photos to sketches - how humans and deep neural networks process objects across different levels of visual abstraction
Source: J Vis. 2022 Feb 7;22(2):4. doi: 10.1167/jov.22.2.4 (PMC8822363; doi:10.1167/jov.22.2.4)
Supplement: Supplement 4 [file jovi-22-2-4_s004.pdf]

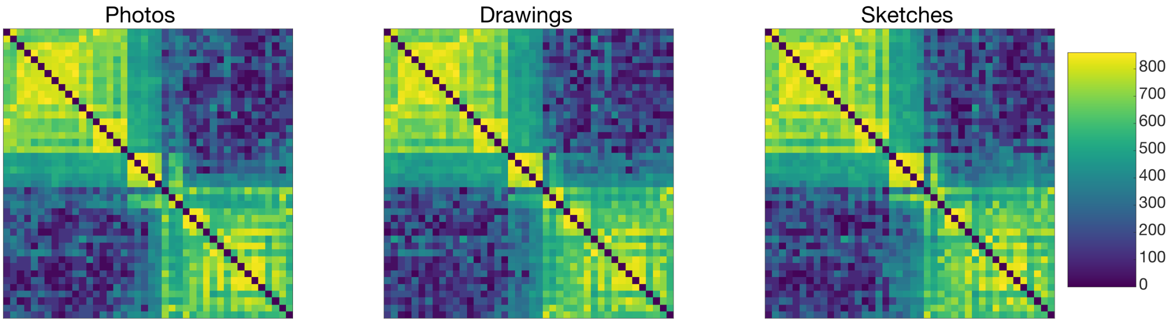

A4. Human behavioral similarity matrices for the three types of depiction. Based on the behavioral similarity judgments in the odd one out similarity task we calculated pairwise similarities between objects as the probability of choosing object x and object y as belonging together regardless of the context imposed by object z in the triplet. For visualization purposes all similarities in one matrix were ranked and sorted according to their superordinate category (manmade/natural).
