# Supplementary material for: From photos to sketches - how humans and deep neural networks process objects across different levels of visual abstraction
Source: J Vis. 2022 Feb 7;22(2):4. doi: 10.1167/jov.22.2.4 (PMC8822363; doi:10.1167/jov.22.2.4)
Supplement: Supplement 5 [file jovi-22-2-4_s005.pdf]

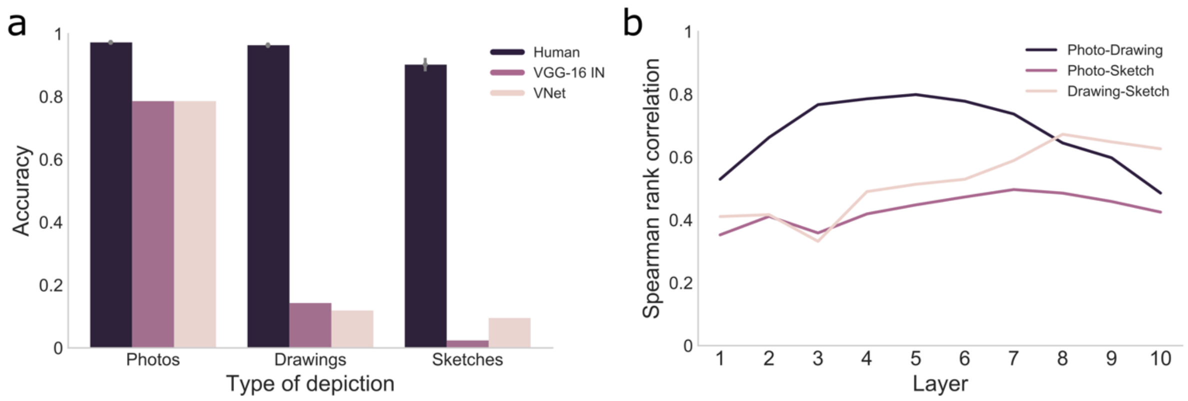

A5. Similarity in processing across levels of visual abstraction in a fully convolutional neural network. a) Top-1 accuracies for vNet for each type of depiction separately in comparison to human and VGG-16 performance. vNet performed similarly as VGG-16 with high performance on photos and poor performance on drawings and sketches. **b)** Representational similarities between types of depiction in vNet. Akin to the experiments with VGG-16 we observed a drop in similarity between representations of photos and both drawings and sketches in the later layers of vNet.
