# Supplementary material for: From photos to sketches - how humans and deep neural networks process objects across different levels of visual abstraction
Source: J Vis. 2022 Feb 7;22(2):4. doi: 10.1167/jov.22.2.4 (PMC8822363; doi:10.1167/jov.22.2.4)
Supplement: Supplement 6 [file jovi-22-2-4_s006.pdf]

Appendix

Classification performance and representational similarities between types of depiction in AlexNet

To demonstrate that the observed results in Experiment 1 are not only specific to the architecture of VGG-16 but can be generalized to other CNN architectures, we evaluated the performance and representational similarities for photos, drawing and sketches in AlexNet pretrained on the ILSVRC2012 dataset (Russakovsky et al., 2015). Analogous to VGG-16, AlexNet also contains a series of convolutional and pooling layers, however differently parametrized in terms of e.g., kernel size, padding, and strides. This series of layers is followed by three fully connected layers at the end of the network.

We first evaluated how the network performed on photos, drawings and sketches in terms of its top-1 accuracy. Analogous to VGG-16 IN, AlexNet showed very high performance on photos and poor performance on drawings and sketches (Fig. A1a). Next, we extracted the activations from all the pooling layers and the first two fully connected layers for all of the images in the three types of depiction and computed RDMs based on these activations for every type of depiction separately. When correlating these RDMs between types of depiction across layers, we found that for the photo-to-drawing and photo-to-sketch comparisons, correlations first increased in pooling layer 1 and 2 but then decreased in the last pooling layer and the fully connected layers (Fig. A1b). For the drawing-sketch comparison the correlations steadily increased across layers. These results closely mirror our findings in VGG-16 IN and therefore suggest that the observed effects are not exclusive to VGG-16 but can be generalized to other similar CNN architectures.

Category-specific information for drawings and sketches across layers in VGG-16

In order to provide additional evidence for the claim that representations in early and intermediate layers in the ImageNet-trained VGG-16 are general enough to support the recognition of drawings and sketches, we asked if we can accurately classify the category of an image based on these representations. For this, we first extracted layer activations from all layers of VGG-16 for all images in the ImageNet-Sketch (Wang et al., 2019) dataset. We then trained Support Vector Machine classifiers (Chang & Lin, 2011) separately for every layer on only the activations from the classes corresponding

to the 42 object categories in our stimulus set to classify the category of the image. In case there was more than one class in ImageNet-Sketch corresponding to a given object category (e.g., multiple dog classes for the object category dog) we randomly sampled 50 of these activations for that given object category. Finally, we evaluated the classifiers on the activation patterns extracted from VGG-16 for our drawing and sketch images separately for both types of depiction. This yielded classification accuracies across layers for both drawings and sketches.

We found that category-specific information increased across the pooling layers for both drawings and sketches but dropped sharply in the fully connected layers (Fig. A3). This further supports the notion that processing in early and intermediate layers in VGG-16 is general enough to allow for the extraction of category-information for both drawings and sketches. In the fully connected layers, however, this category-specific information was largely lost, in line with a shift in representations of drawings and sketches possibly due to biases for the statistics of natural images in the network.

Representational similarities between types of depiction in a fully convolutional neural network

In order to test the role of fully connected layers in representational similarities of object images across levels of abstraction, we analyzed the fully convolutional neural network vNet (Mehrer et al., 2021). The architecture of vNet includes 10 layers with increasing kernel size mirroring the increase of average receptive field sizes along areas of the human ventral stream followed by a classification layer. Each of the layers in the network entails a convolution operation, dropout, max-pooling, group-norm and a ReLU nonlinearity (except layers 1,2,5,6 which do not include max-pooling). Importantly, in contrast to VGG-16, vNet does not have fully connected layers apart from the final classification layer. The network was trained on the ILSVRC2012 (Russakovsky et al., 2015) dataset such as the network in Experiment 1.

Analogous to the procedure in experiments 1 to 3, we first obtained the top-1 accuracies for the stimuli in each type of depiction separately. VNet showed high accuracy on photos (*M*(Photos)=0.79) and a drop in performance for drawings and sketches (*M*(Drawings)=0.12, *M*(Sketches)=0.1), similar to the results in Experiment 1. Subsequently, we extracted the activations for our stimuli from all of the layers in vNet

and computed RDMs based on the activations for all the types of depiction and layers separately. Finally, we computed the Spearman rank correlation between the lower triangular values of the RDMs of the different types of depiction for each of the layers. We found that representational similarities between photos and drawings increased in the early layers and reached a peak in layer 5 after which there was a drop in representational similarity (Fig. A5). For the photo-to-sketch similarity a similar pattern was observed with an increase in similarity in early layers and a drop in the later layers after a peak in layer 7. Further, for the drawing-to-sketch correlation the similarity increased steadily up until the last layer in which the similarity dropped slightly compared to the penultimate layer.

In sum, the representational similarities between photos and abstracted types of depiction followed a comparable pattern to what was observed in the ImageNet-trained VGG-16 in Experiment 1 with a drop in similarity in the later layers close to the classification layer. Taken together, this indicates that the poor performance on drawings and sketches and the drop in representational similarity in VGG-16 between types of depiction cannot be fully explained by the presence of fully connected layers.
